# Supplementary material for: Leafhopper salivary vitellogenin mediates virus transmission to plant phloem
Source: Nat Commun. 2024 Jan 2;15:3. doi: 10.1038/s41467-023-43488-5 (PMC10762104; doi:10.1038/s41467-023-43488-5)
Supplement: Supplementary file 1 — Supplementary information [file 41467_2023_43488_MOESM1_ESM.pdf]

SUPPLEMENTARY INFORMATION

Leafhopper salivary vitellogenin mediates virus transmission to plant phloem

Wang et al.

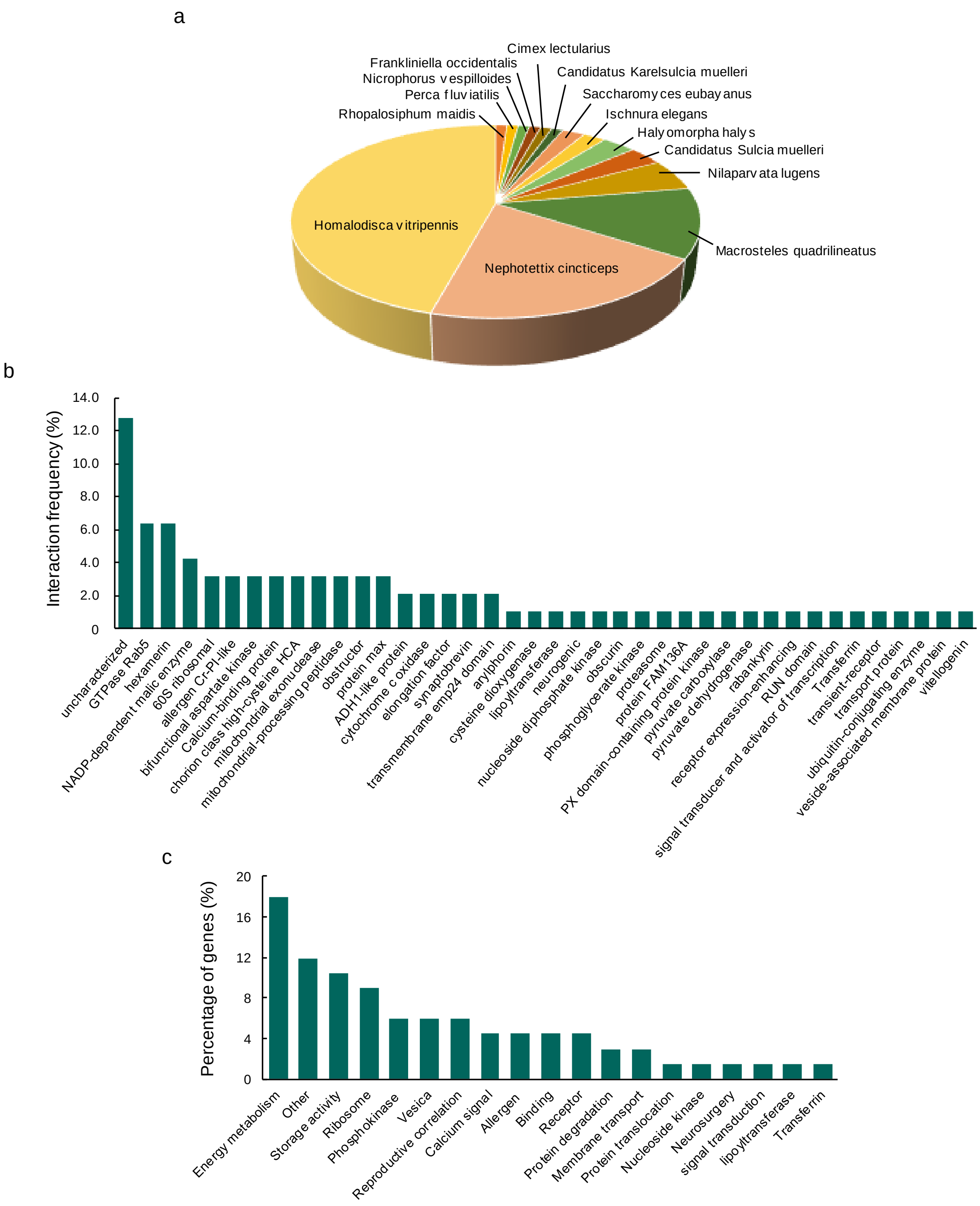

Supplementary Figure 1 Analysis of putative interactors of NcVg2 in Y2H screening. a Species distribution percentage of putative interactors of NcVg2. Each pie presents the species hit by the putative interactors in BLASTX analyses. **b** Interaction frequency of more than 40 putative interactors of *N. cincticeps* from the Y2H system. **c** GO categories of the putative interactors of *N. cincticeps* on molecular function. Nineteen functional groups of putative interactors with annotation were identified.

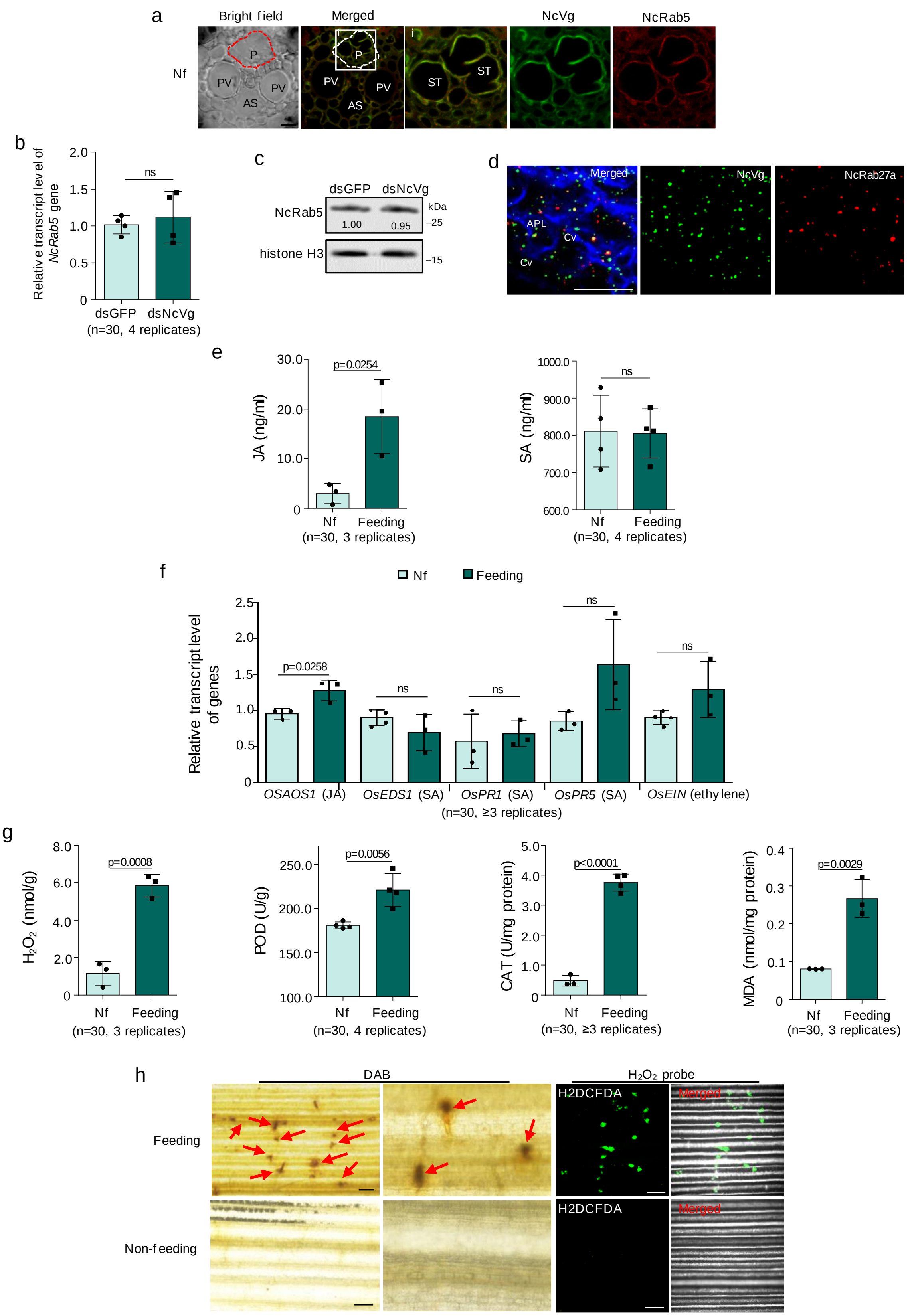

Supplementary Figure 2 Nonviruliferous leafhoppers feeding increasing levels of H_2O_2

and related metabolism. a Distribution of NcVg and NcRab5 in rice phloem of which plant separated from leafhoppers, as determined by immunofluorescence microscopy. Sections of rice plants were immunolabeled with NcVg-FITC (green) and NcRab5-rhodamine (red). Panel i is the enlarged image of the boxed area. P, phloem; AS, air space; PV, pitted vessel; ST, sieve tube. Bars, 10 μm. **b** and **c** Knockdown of *NcVg* expression causing limited effect on NcRab5 expression in salivary glands. Data in **b** are from salivary glands of 30 dsNcVg- or dsGFP-treated leafhoppers. The proteins from salivary glands of 50 dsNcVg- or dsGFP-treated leafhoppers were detected using NcRab5- or histone H3-specific antibody in western blot assays. The relative intensities of the bands are shown. **d** Distribution of NcVg and NcRab27a in cavities and cytoplasm of salivary gland, as determined by immunofluorescence microscopy. Salivary glands of nonviruliferous leafhoppers were fixed, immunostained with NcVg-FITC (green), NcRab27a-rhodamine (red) and actin dye phalloidin-Alexa Fluor 647 carboxylic acid (blue). APL, apical plasmalemma. Cv, cavity. Bars, 10 μm. **e** and **f** Nonviruliferous leafhoppers feeding causing limited effect on the content of JA and SA (**e**) as well as the expression of related genes of JA, SA, and ethylene (**f**), as determined by mass spectrometer and RT-qPCR assays. **g** Nonviruliferous leafhoppers feeding increasing contents of H_2O_2 and MDA, as well as activity of CAT and POD in rice seedlings. Nf, non-feeding. Ns, not significant. **h** Location and accumulation of H_2O_2 in leaves exposed to leafhoppers for 12 hours, as determined by DAB or H2DCF-DA staining. One leaf of rice seedling exposed to 5 leafhoppers for 12 hours was stained with DAB or H2DCF-DA. Bars, 200 μm. All data represent at least 3 biological replicates. Data in **e**, **f** and **g** are from 1 rice seedling exposed or not exposed to 30 nonviruliferous leafhoppers for 12 hours. Means (± SD) in **b**, **e**, **f** and **g** are shown and analyzed using two-tailed t-test.

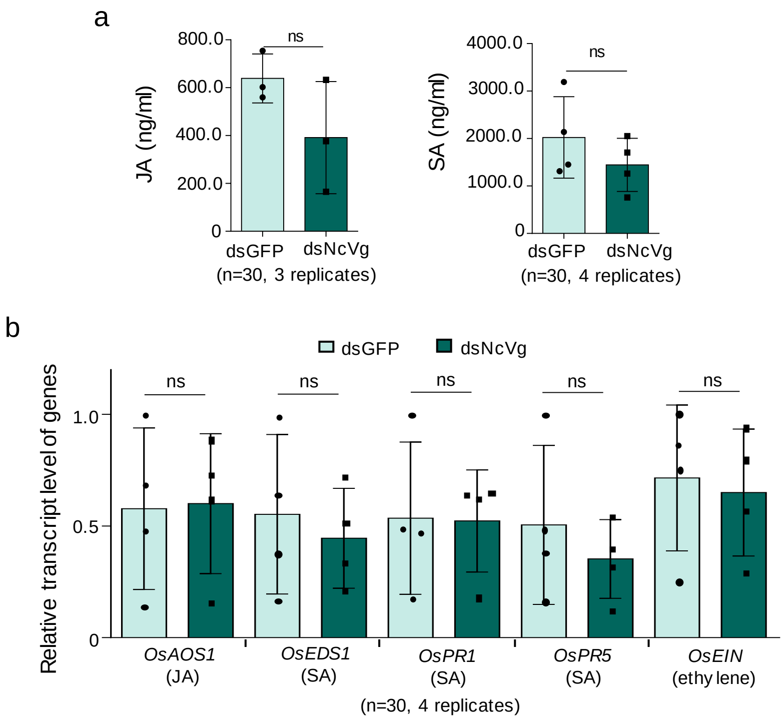

Supplementary Figure 3 Effects of *NcVg* knockdown on contents of JA and SA (a), as well as gene expression of related genes of JA, SA and ethylene (b), determined by mass spectrometer and RT-qPCR assays. Means (± SD) are shown from 1 rice seedling exposed to 30 dsNcVg- or dsGFP-treated nonviruliferous leafhoppers for 12 hours, and represent 4 biological replicates (two-tailed t-test). Ns, not significant.

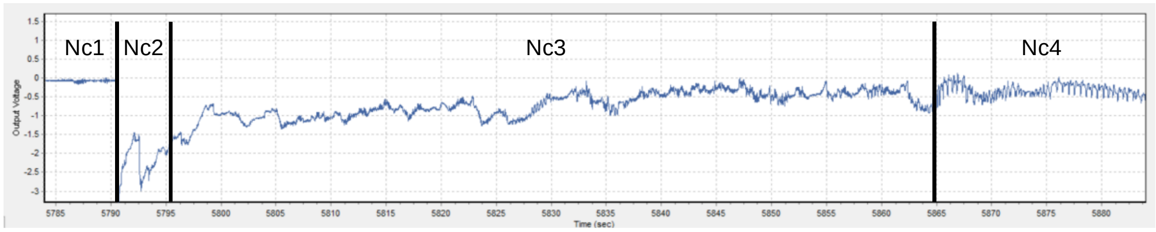

Supplementary Figure 4 Characterization of the EPG waveforms produced by *N. cincticeps* feeding on rice plants. Nc1, no probing. Nc2, stylet penetration into host. Nc3, stylet pathway and salivation. Nc4, stylets in phloem and xylem tissue. Data represent at least 3 biological replicates.

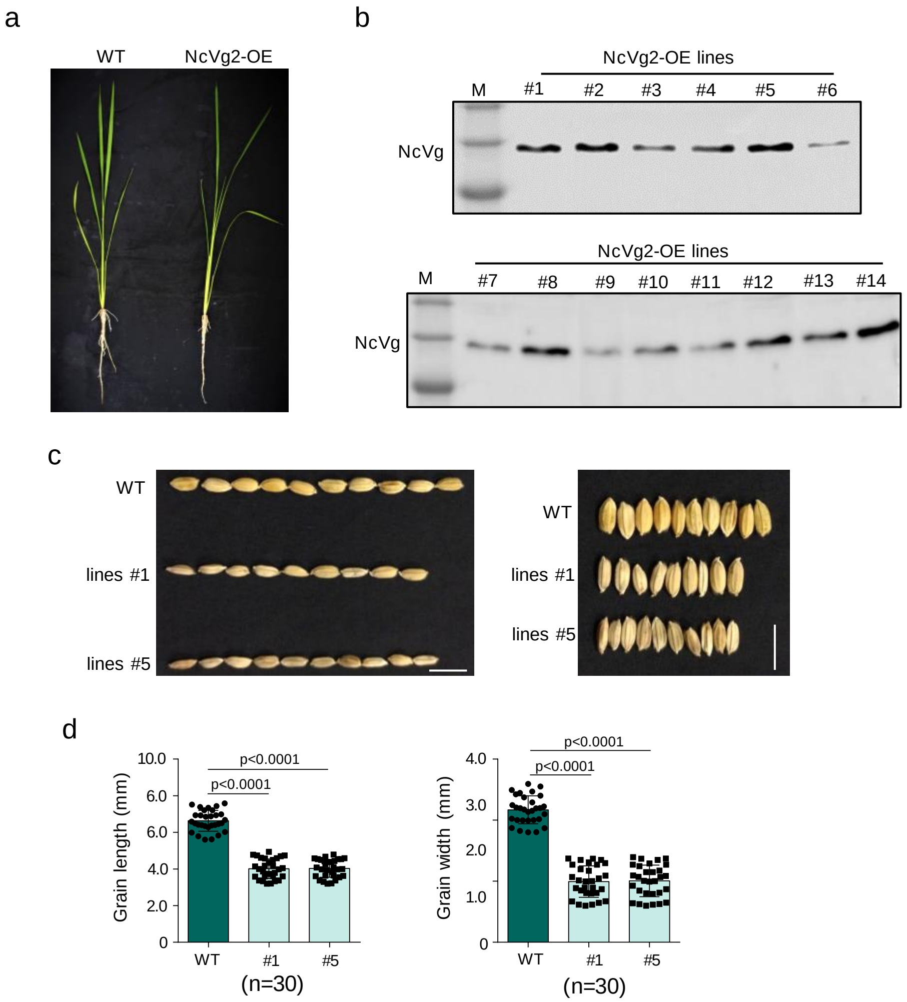

Supplementary Figure 5 Characterization of basic properties of NcVg2-OE transgenic plants. a Comparison of phenotypic traits between WT and NcVg2-OE transgenic plants. **b** NcVg2 expression in NcVg2-OE transgenic plants from #1 to #14, as determined by western blot assays. The proteins were detected by using NcVg-specific antibody in western blot assays. **c** Grain phenotypes of lines #1 and #5, as well as WT in the background of *O. sativa* L.ssp. *Japonica*, variety *Nipponbare*. Bars, 10 mm. Data in **a**, **b** and **c** represent 3 biological replicates. **d** Measurement of grain length and width of the WT, #1 and #5 lines. Means (± SD) are shown and analyzed using two-tailed t-test.

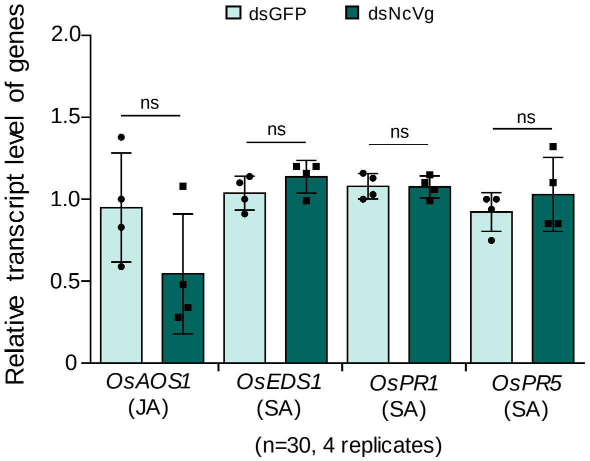

Supplementary Figure 6 Effects of *NcVg* knockdown in viruliferous leafhoppers on the expression of genes related JA, SA, and ethylene, as determined by RT-qPCR assays. Means (± SD) are shown from 1 rice seedlings exposed to 30 dsNcVg- or dsGFP-treated viruliferous leafhoppers, and represent 4 replicates. Data are analyzed using two-tailed t-test. Ns, not significant.

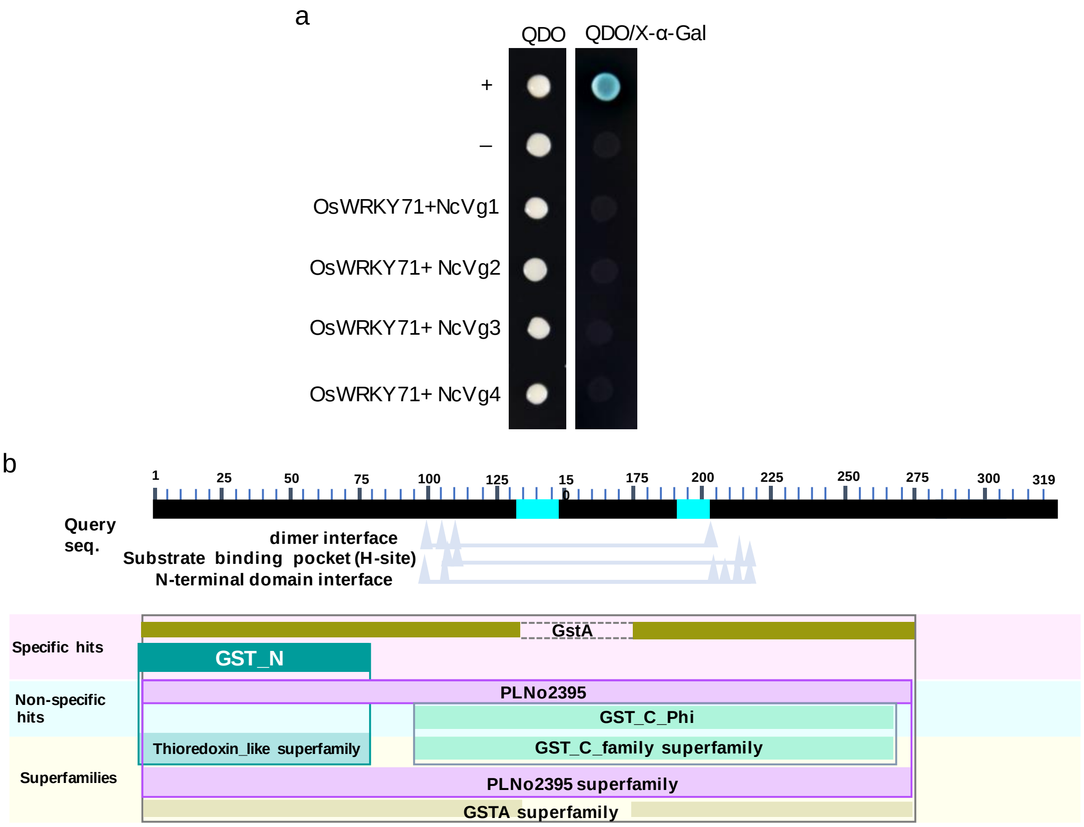

Supplementary Figure 7 Interaction of NcVg with OsWRKY71 and conserved domain of OsGSTF12. a Y2H assays showing no interaction of NcVg with OsWRKY71. Transformants are labeled as follows: OsWRKY71+NcVg1, pGBKT7-OsWRKY71/pGADT7-NcVg1; OsWRKY71+NcVg2, pGBKT7-OsWRKY71/pGADT7-NcVg2; OsWRKY71+NcVg3, pGBKT7-OsWRKY71/pGADT7-NcVg3; OsWRKY71+NcVg4, pGBKT7-OsWRKY71/pGADT7-NcVg4; +, positive control; –, negative control. QDO, SD/-Trp-Leu-His-Ade medium. **b** Schematic representation of OsGSTF12 conserved domain.

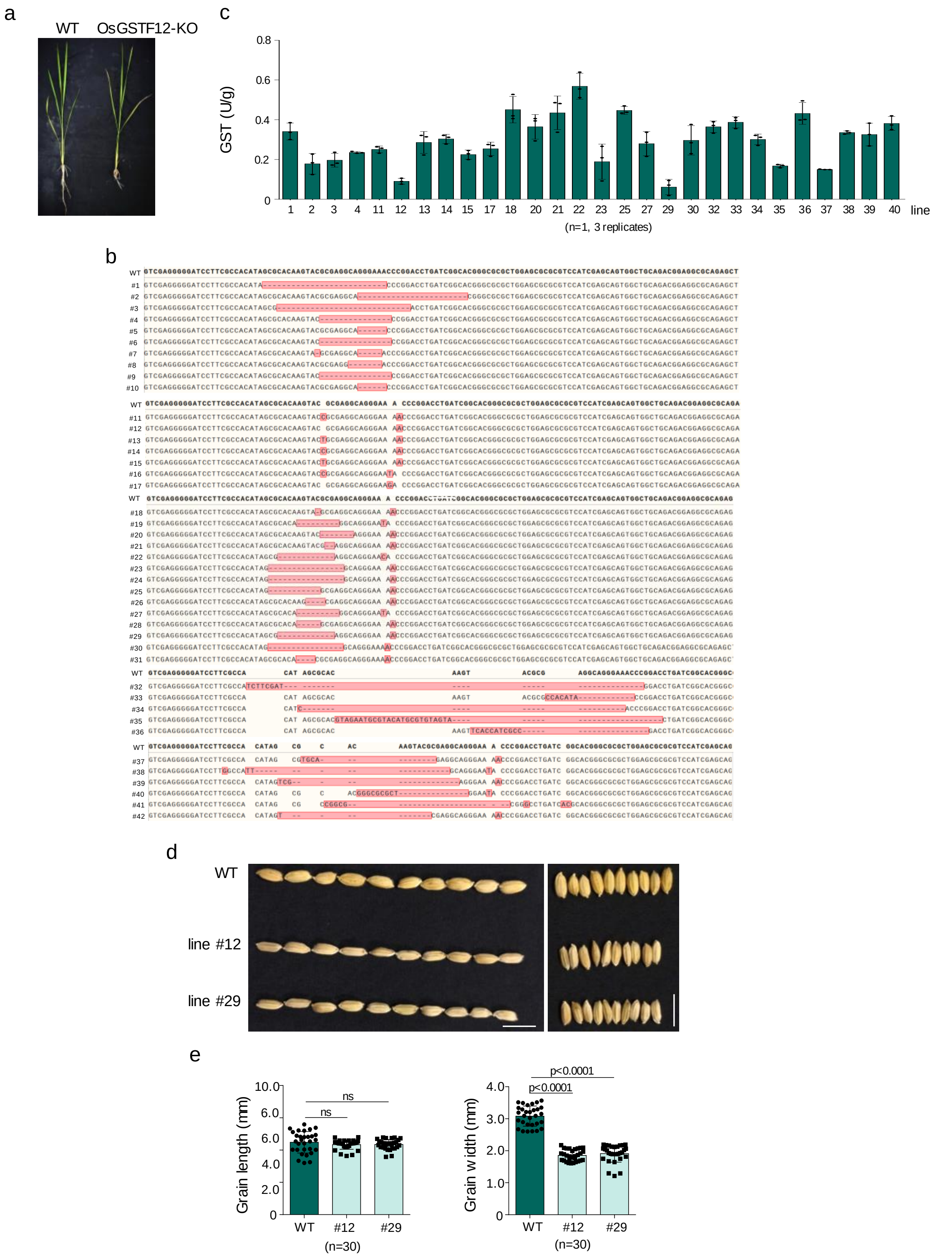

Supplementary Figure 8 Characterization of basic properties of OsGSTF12-KO transgenic plants. a Comparison of phenotypic traits between WT and OsGSTF12-KO transgenic plants. **b** OsGSTF12 mutation in the T_0 generation transgenic rice plants. WT,

wild‐type sequence. ‐, deleted nucleotides. Sequences in red box indicate insertions or substitutions. **c** Activity of GST in OsGSTF12-KO transgenic plants. **d** Grain phenotypes of lines #12 and #29, as well as WT in the background of *O. sativa* L.ssp. *Japonica*, variety *Nipponbare*. Bars, 10 mm. Data in **a**, **c** and **d** represent 3 biological replicates. **e** Measurement of grain length and width of the WT, #12 and #29 lines. Ns, not significant. Means (± SD) in **c** and **e** are shown and analyzed using two-tailed t-test.

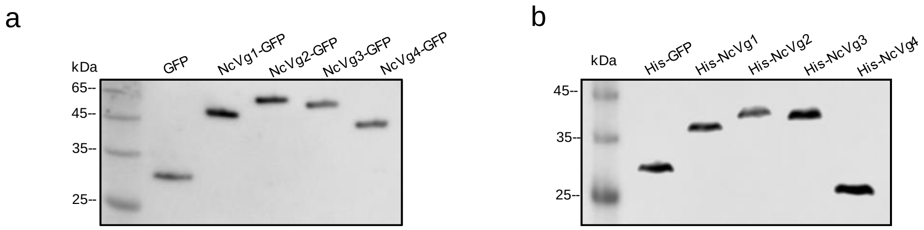

Supplementary Figure 9 Expression of NcVg1 to NcVg4 *in vitro*, as determined by western blot analysis. a Transient expression of NcVg1 to NcVg4 in *N. benthamiana*. Proteins were detected using GFP antibodies in western blot assays. **b** Prokaryotic expression of NcVg1 to NcVg4 in *E. coli* strain BL21 cells. Proteins were detected using antibodies against 6×His tag in western blot assays. Data represent 3 biological replicates.

Supplementary Table 1 BLASTX search of Genbank database for putative interactors of NcVg2 in the Y2H screening.

NO.	Accession	Protein	Species	Identities (%)
1	YP_009136185.1	cytochrome c oxidase	*N. cincticeps*	99
2	MW266984.1	GTPase Rab5	*N. cincticeps*	100
3	XP_054288952.1	arylphorin	*Macrosteles quadrilineatus*	83
4	XP_054281109.1	RUN domain	*M. quadrilineatus*	91

5	XP_054272432.1	proteasome	*M. quadrilineatus*	78
6	XP_054271750.1	allergen Cr-PI-like	*M. quadrilineatus*	76
7	XP_054266746.1	NADP-dependent malic enzyme	*M. quadrilineatus*	83
8	XP_046684145.1	rabankyrin	*Homalodisca vitripennis*	86
9	XP_046683184.1	transmembrane emp24 domain	*H. vitripennis*	88
10	XP_046683179.1	protein FAM136A	*H. vitripennis*	79
11	XP_046683049.1	neurogenic	*H. vitripennis*	83
12	XP_046682950.1	cysteine dioxygenase	*H. vitripennis*	84
13	XP_046678504.1	chorion class high-cysteine HCA	*H. vitripennis*	85
14	XP_046676949.1	nucleoside diphosphate kinase	*H. vitripennis*	79
15	XP_046673692.1	transport protein	*H. vitripennis*	87
16	XP_046673290.1	60S ribosomal	*H. vitripennis*	88
17	XP_046673288.1	synaptobrevin	*H. vitripennis*	87
18	XP_046672836.1	vitellogenin	*H. vitripennis*	84
19	XP_046671720.1	lipoyltransferase	*H. vitripennis*	88
20	XP_046669576.1	obstructor	*H. vitripennis*	67
21	XP_046671688.1	hexamerin	*H. vitripennis*	55
22	XP_046663903.1	mitochondrial exonuclease	*H. vitripennis*	87
23	XP_046662026.1	PX domain-containing protein kinase	*H. vitripennis*	76
24	XP_046661294.1	receptor expression-enhancing	*H. vitripennis*	84
25	XP_046661139.1	obscurin	*H. vitripennis*	74
26	XP_046382644.1	elongation factor	*Ischnura elegans*	86
27	XP_039633766.1	signal transducer and activator of transcription	*Perca fluviatilis*	77
28	XP_039293902.1	pyruvate carboxylase	*Nilaparvata lugens*	75
29	XP_039282363.1	protein max	*N. lugens*	78

30	XP_026823020.1	vesicle-associated membrane protein	*Rhopalosiphum maidis*	81
31	XP_026287297.1	ubiquitin-conjugating enzyme	*Frankliniella occidentalis*	81
32	XP_018221708.1	ADH1-like protein	*Saccharomyces eubayanus*	75
33	XP_017781146.1	phosphoglycerate kinase	*Nicrophorus vespilloides*	92
34	XP_014286882.1	mitochondrial-processing peptidase	*Halyomorpha halys*	83
35	XP_014240468.1	transient-receptor	*Cimex lectularius*	80
36	WP_196767579.1	bifunctional aspartate kinase	*Candidatus Sulcia muelleri*	75
37	WP_020931738.1	pyruvate dehydrogenase	*C. Karelsulcia muelleri*	78
38	G9M8X1.1	calcium-binding protein	*N. cincticeps*	95
39	BAQ94504.1	Transferrin	*N. cincticeps*	88

Uncropped blots used in the main figures

Fig. 1b

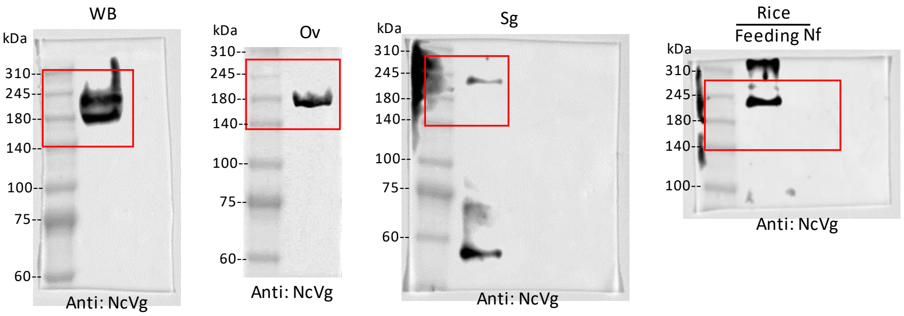

Fig. 1d

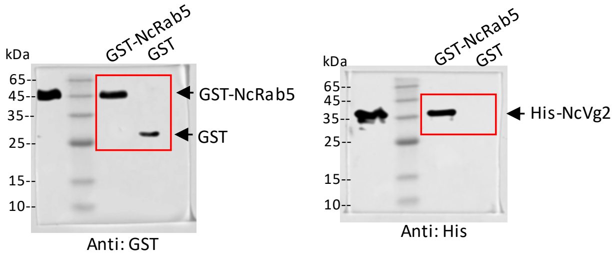

Fig. 1f

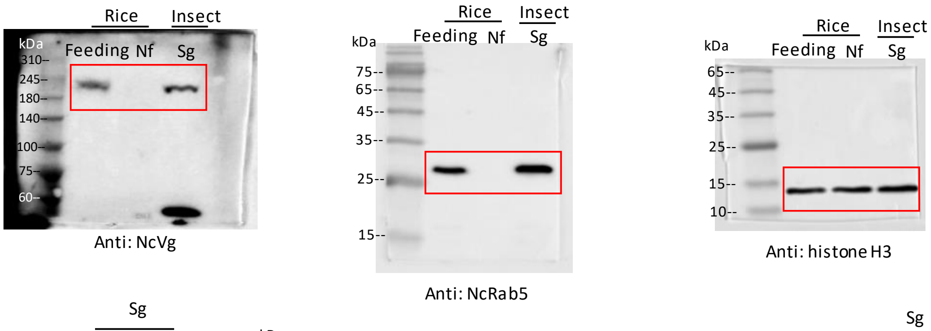

Fig. 1h

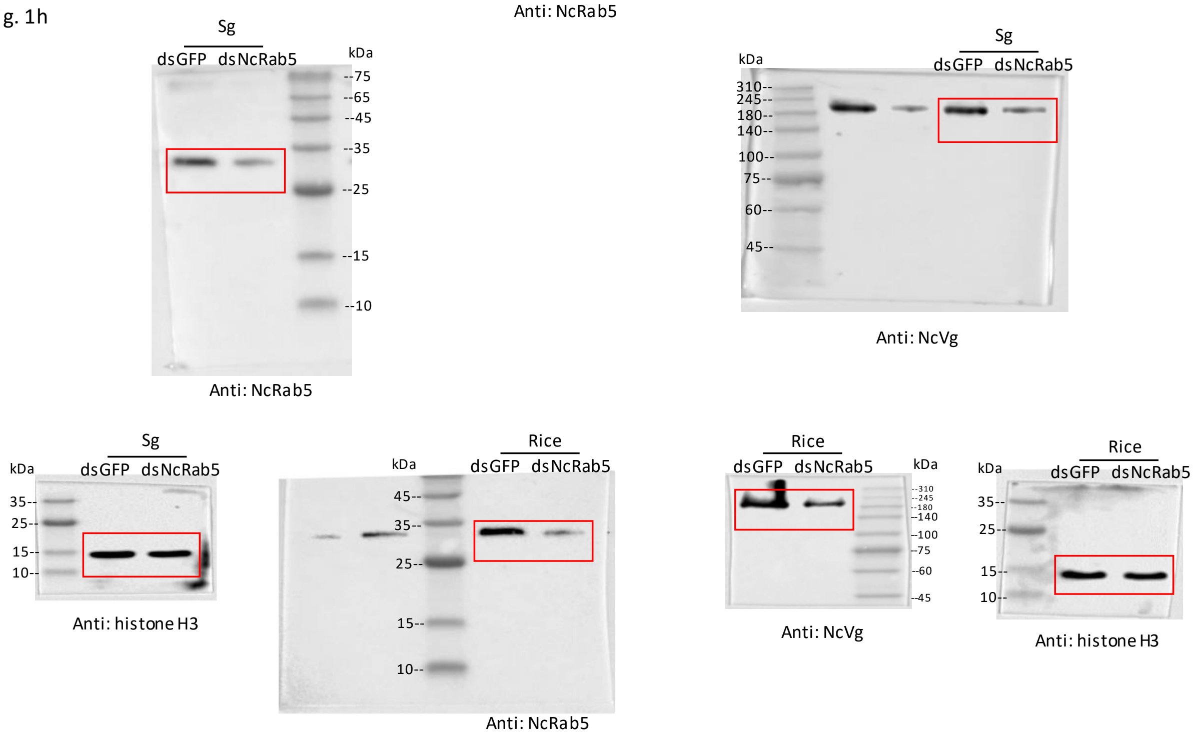

Fig. 1j

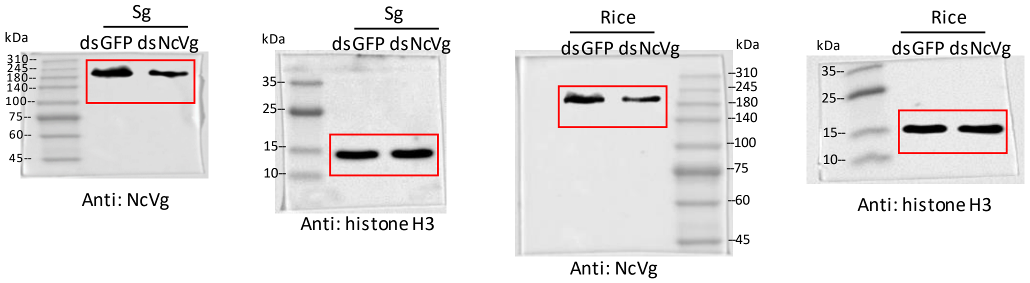

Sg
dsGFP dsNcVg
kDa
310
245
180
140
100
75
60
45
Anti: NcVg
Sg
dsGFP dsNcVg
kDa
35
25
15
10
Anti: histone H3
Rice
dsGFP dsNcVg
kDa
310
245
180
140
100
75
60
45
Anti: NcVg
Rice
dsGFP dsNcVg
kDa
35
25
15
10
Anti: histone H3

Fig. 2b

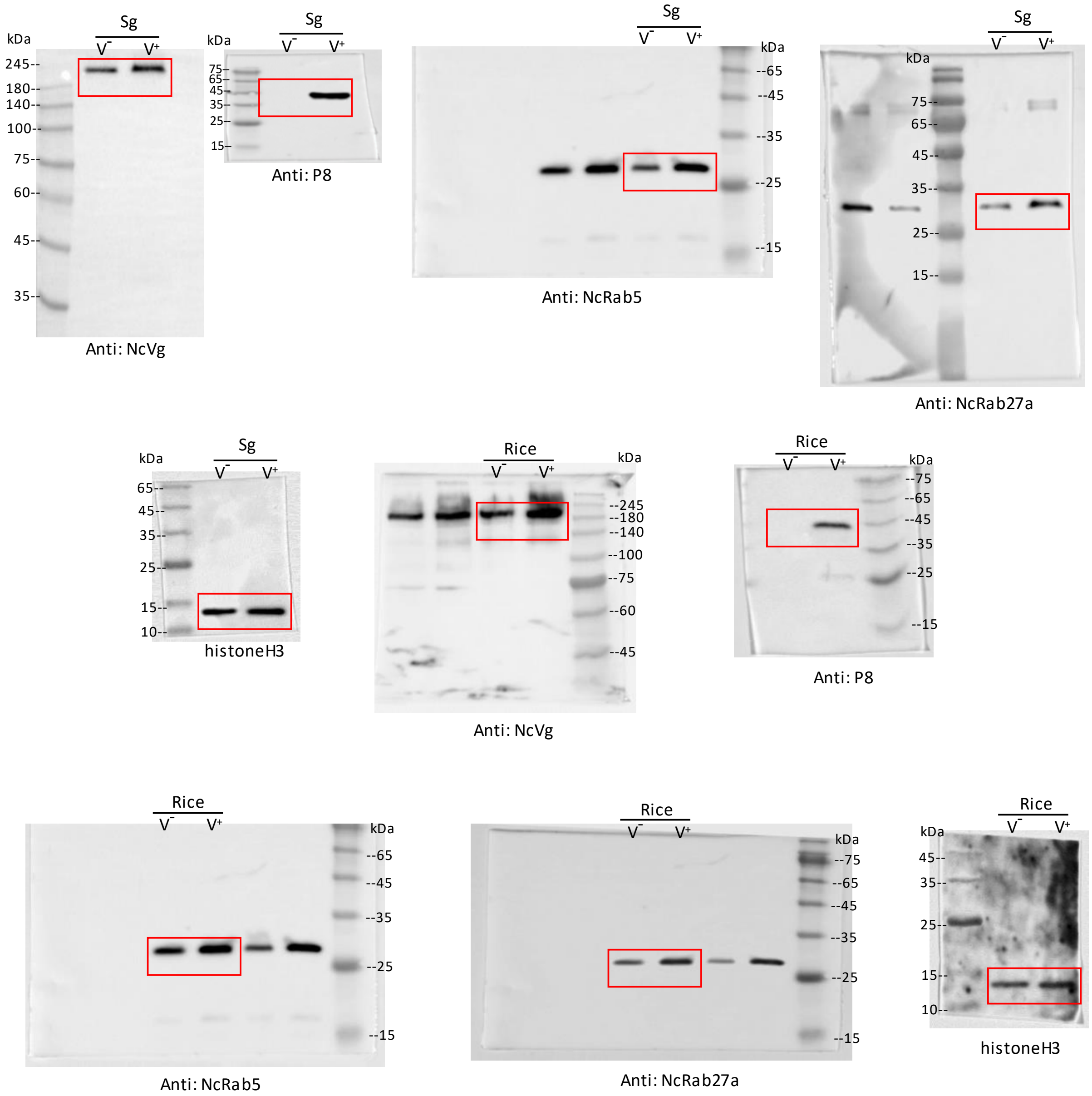

Sg
V⁻ V⁺
kDa
245
180
140
100
75
60
45
35
Anti: NcVg
Sg
V⁻ V⁺
kDa
75
65
45
35
25
15
Anti: P8
Sg
V⁻ V⁺
kDa
65
45
35
25
15
Anti: NcRab5
Sg
V⁻ V⁺
kDa
75
65
45
35
25
15
Anti: NcRab27a
Sg
V⁻ V⁺
kDa
65
45
35
25
15
10
histoneH3
Rice
V⁻ V⁺
kDa
245
180
140
100
75
60
45
Anti: NcVg
Rice
V⁻ V⁺
kDa
75
65
45
35
25
15
Anti: P8
Rice
V⁻ V⁺
kDa
65
45
35
25
15
Anti: NcRab5
Rice
V⁻ V⁺
kDa
75
65
45
35
25
15
Anti: NcRab27a
Rice
V⁻ V⁺
kDa
45
35
25
15
10
histoneH3

Fig. 2f

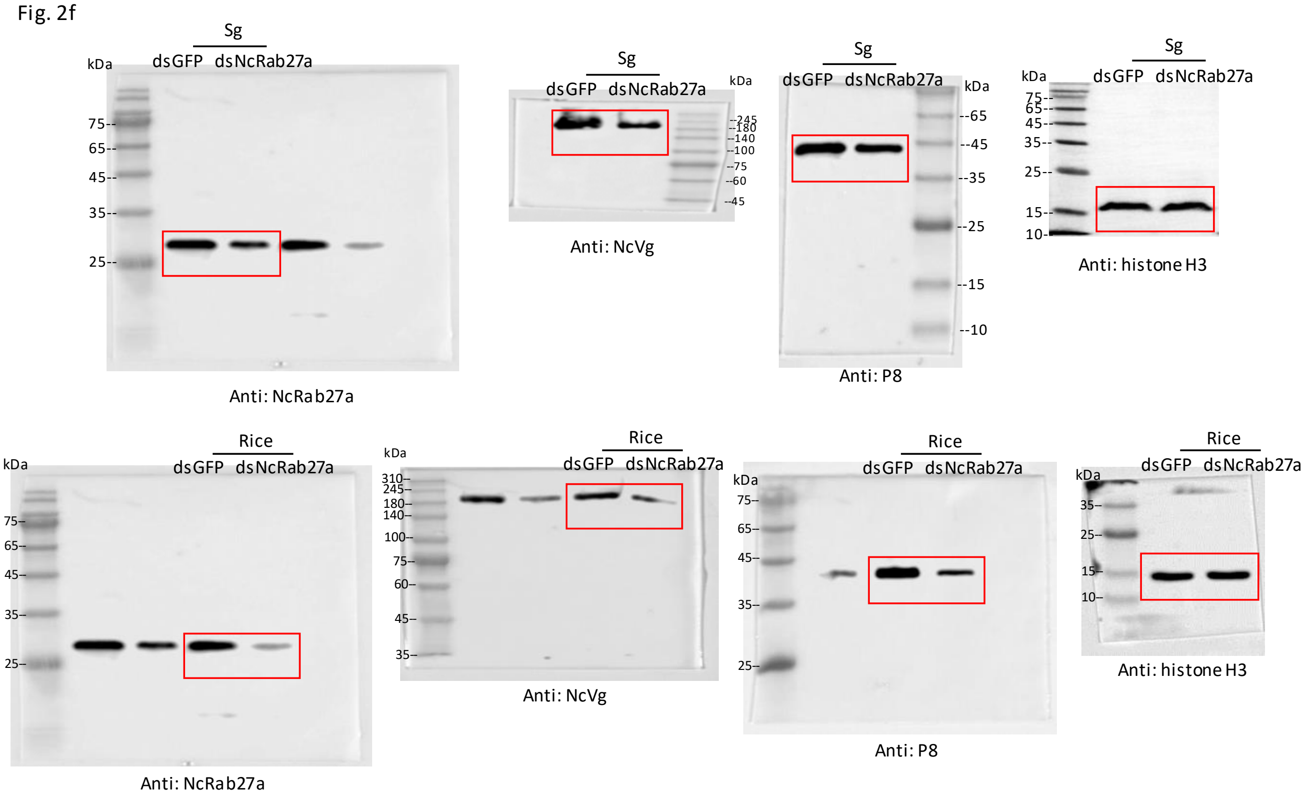
Sg
dsGFP dsNcRab27a
kDa
75
65
45
35
25
Anti: NcRab27a
Sg
dsGFP dsNcRab27a
kDa
245
180
140
100
75
60
45
Anti: NcVg
Sg
dsGFP dsNcRab27a
kDa
65
45
35
25
15
10
Anti: P8
Sg
dsGFP dsNcRab27a
kDa
75
65
45
35
25
15
10
Anti: histone H3
Rice
dsGFP dsNcRab27a
kDa
75
65
45
35
25
Anti: NcRab27a
Rice
dsGFP dsNcRab27a
kDa
310
245
180
140
100
75
60
45
35
Anti: NcVg
Rice
dsGFP dsNcRab27a
kDa
75
65
45
35
25
Anti: P8
Rice
dsGFP dsNcRab27a
kDa
35
25
15
10
Anti: histone H3

Fig. 2g

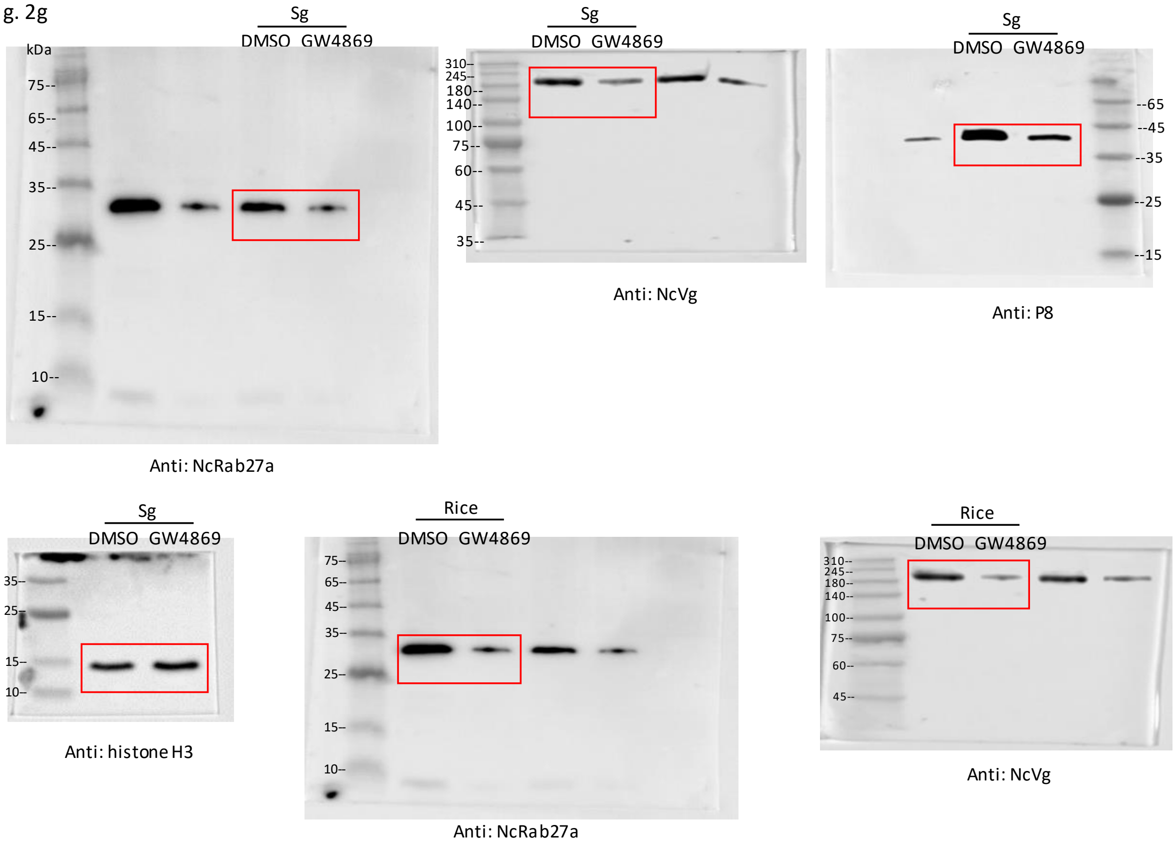
Sg
DMSO GW4869
kDa
75
65
45
35
25
15
10
Anti: NcRab27a
Sg
DMSO GW4869
310
245
180
140
100
75
60
45
35
Anti: NcVg
Sg
DMSO GW4869
65
45
35
25
15
Anti: P8
Sg
DMSO GW4869
35
25
15
10
Anti: histone H3
Rice
DMSO GW4869
75
65
45
35
25
15
10
Anti: NcRab27a
Rice
DMSO GW4869
310
245
180
140
100
75
60
45
Anti: NcVg

Fig. 2g

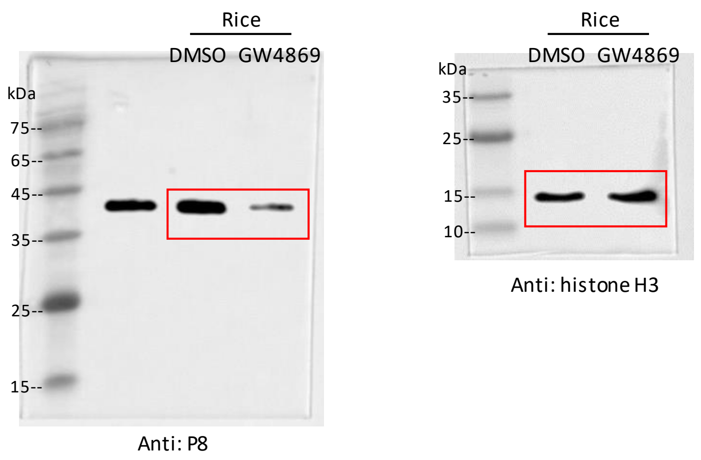

Fig. 3a

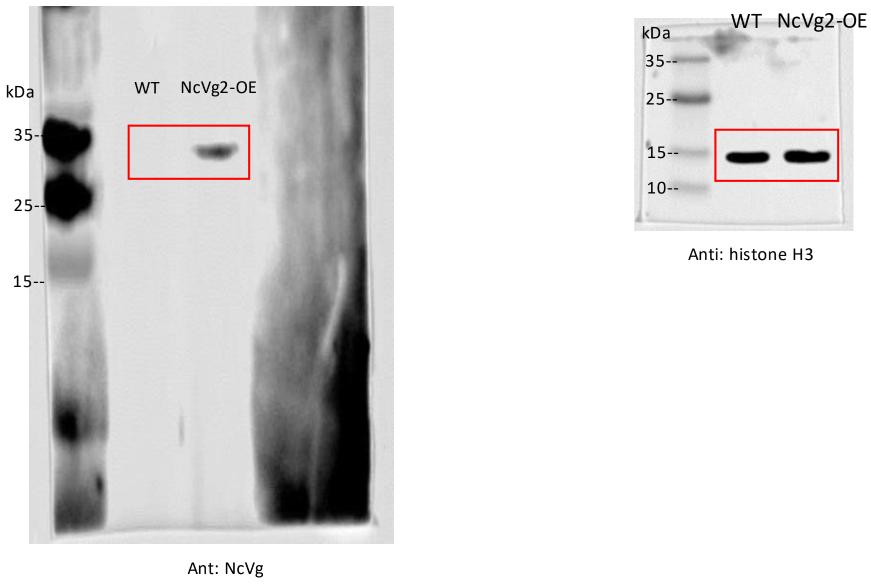

Fig. 5b

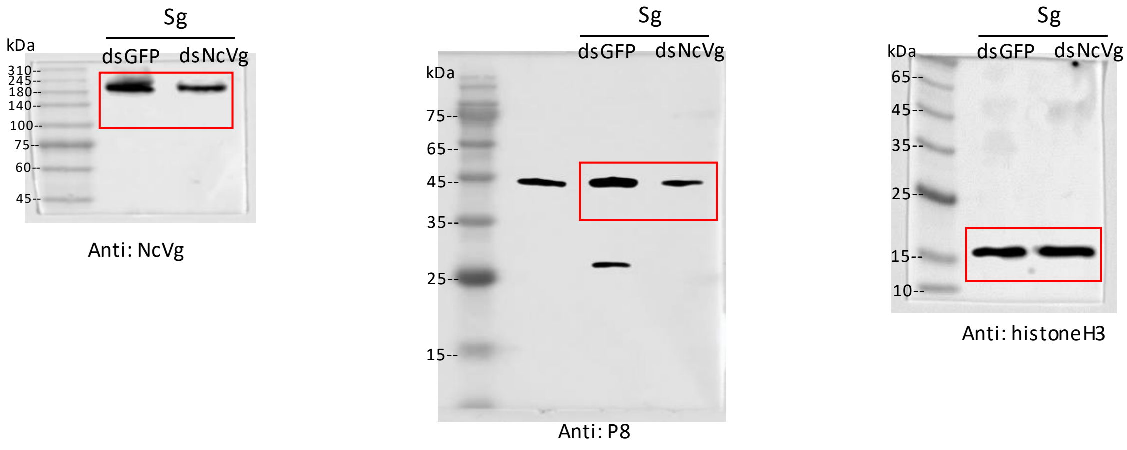

Fig. 5b

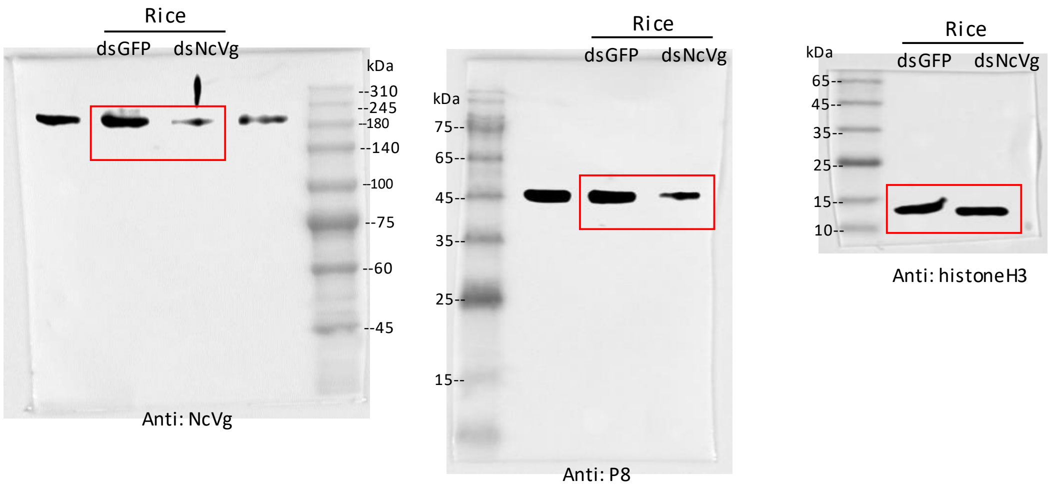

Fig. 6g

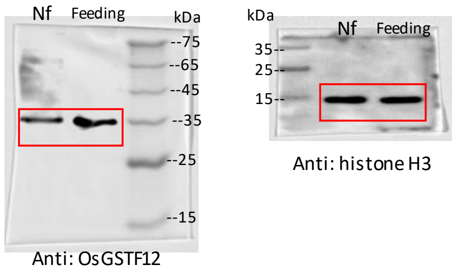

Fig. 6l

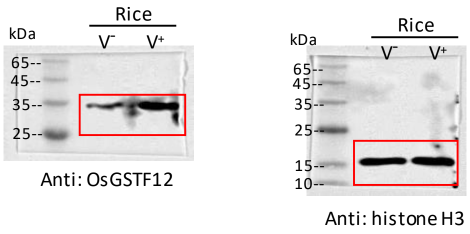

Fig. 6k

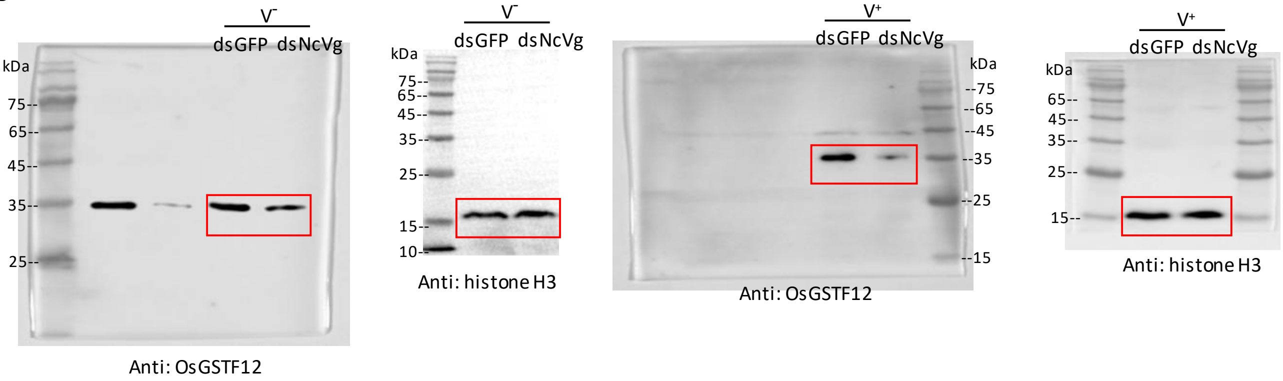

Fig. 7e

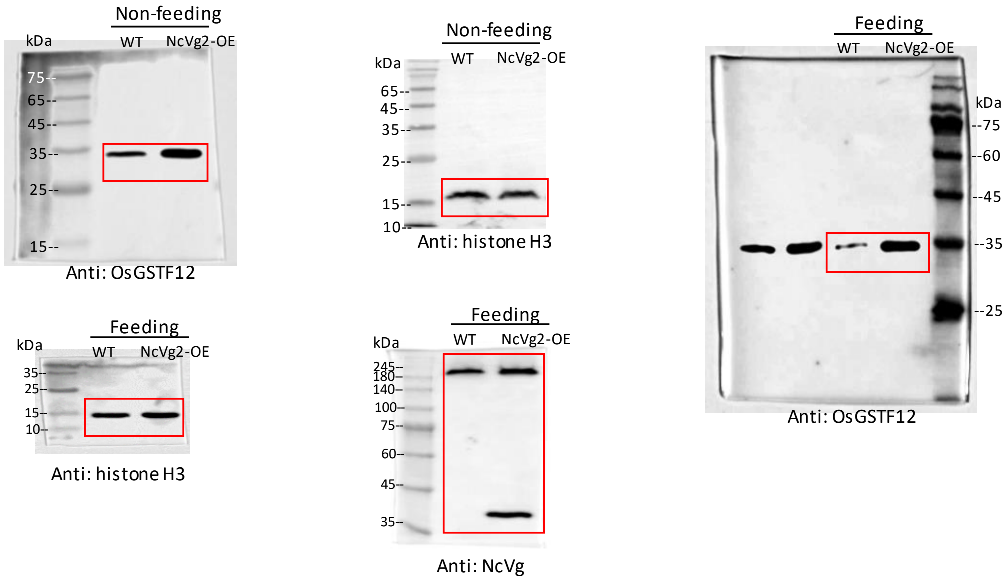

Fig. 8a

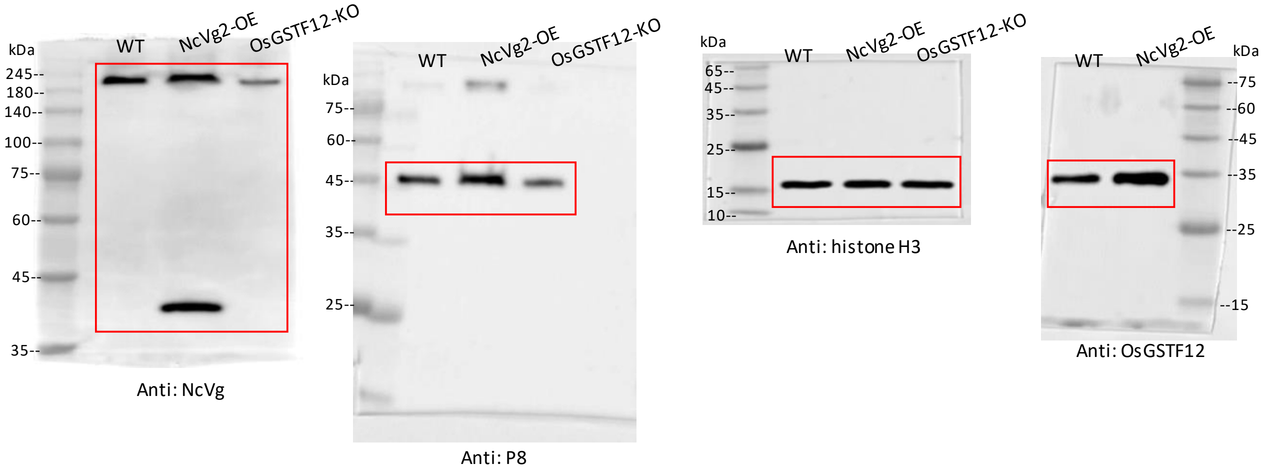

Fig. S2c

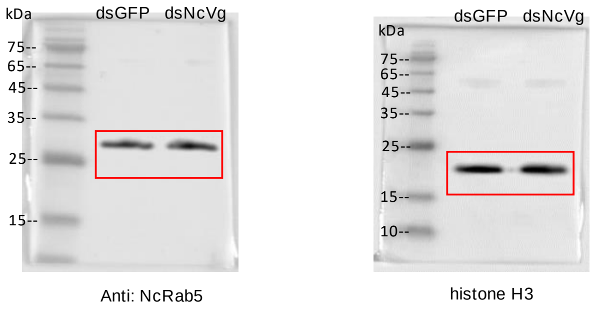

Fig. S5b

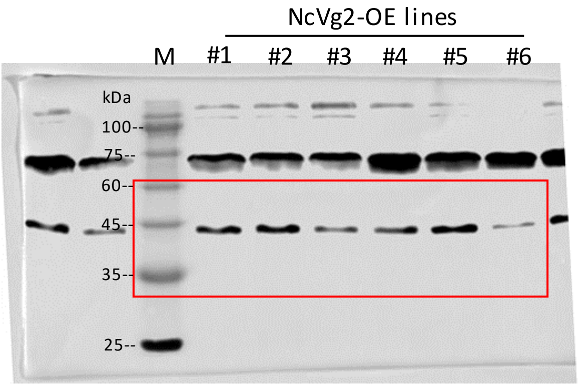

Anti: NcVg

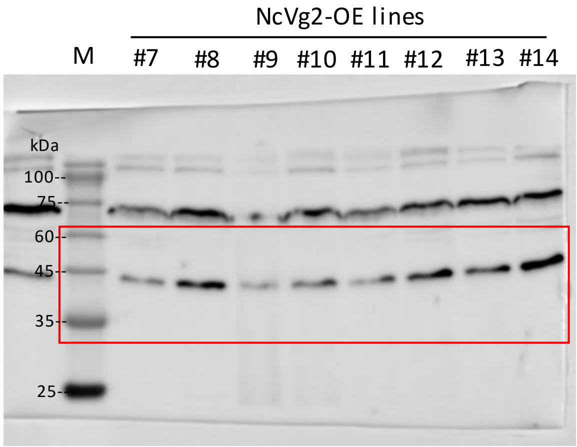

Anti: NcVg

Fig. S9a

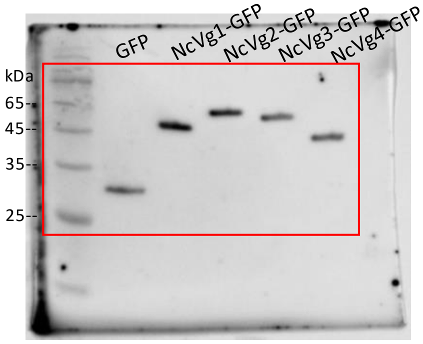

Anti: GFP

Fig. S9b

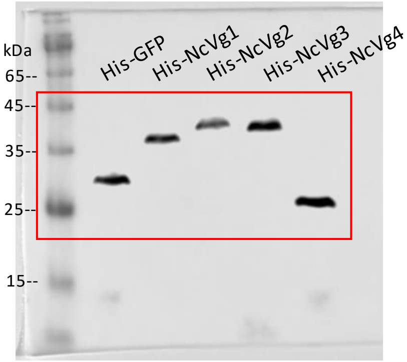

Anti: His
