# Supplementary material for: Leafhopper salivary vitellogenin mediates virus transmission to plant phloem
Source: Nat Commun. 2024 Jan 2;15:3. doi: 10.1038/s41467-023-43488-5 (PMC10762104; doi:10.1038/s41467-023-43488-5)
Supplement: Supplementary file 3 — Reporting Summary [file 41467_2023_43488_MOESM3_ESM.pdf]

nature portfolio

Corresponding author(s): Qian Chen

Last updated by author(s): Oct 21, 2023

Reporting Summary

Nature Portfolio wishes to improve the reproducibility of the work that we publish. This form provides structure for consistency and transparency in reporting. For further information on Nature Portfolio policies, see our Editorial Policies and the Editorial Policy Checklist.

Statistics

For all statistical analyses, confirm that the following items are present in the figure legend, table legend, main text, or Methods section.

n/a	Confirmed	
☐	☒	The exact sample size (*n*) for each experimental group/condition, given as a discrete number and unit of measurement
☐	☒	A statement on whether measurements were taken from distinct samples or whether the same sample was measured repeatedly
☐	☒	The statistical test(s) used AND whether they are one- or two-sided<br>*Only common tests should be described solely by name; describe more complex techniques in the Methods section.*
☒	☐	A description of all covariates tested
☒	☐	A description of any assumptions or corrections, such as tests of normality and adjustment for multiple comparisons
☒	☐	A full description of the statistical parameters including central tendency (e.g. means) or other basic estimates (e.g. regression coefficient) AND variation (e.g. standard deviation) or associated estimates of uncertainty (e.g. confidence intervals)
☐	☒	For null hypothesis testing, the test statistic (e.g. *F*, *t*, *r*) with confidence intervals, effect sizes, degrees of freedom and *P* value noted<br>*Give P values as exact values whenever suitable.*
☒	☐	For Bayesian analysis, information on the choice of priors and Markov chain Monte Carlo settings
☒	☐	For hierarchical and complex designs, identification of the appropriate level for tests and full reporting of outcomes
☒	☐	Estimates of effect sizes (e.g. Cohen's *d*, Pearson's *r*), indicating how they were calculated

Our web collection on statistics for biologists contains articles on many of the points above.

Software and code

Policy information about availability of computer code

Data collection	The gene sequences of insect or plant are collected from Genbank.
Data analysis	All quantitative data presented in figures were analyzed using two-tailed t-tests in GraphPad Prism 7 software.

For manuscripts utilizing custom algorithms or software that are central to the research but not yet described in published literature, software must be made available to editors and reviewers. We strongly encourage code deposition in a community repository (e.g. GitHub). See the Nature Portfolio guidelines for submitting code & software for further information.

Data

Policy information about availability of data

All manuscripts must include a data availability statement. This statement should provide the following information, where applicable:

- Accession codes, unique identifiers, or web links for publicly available datasets
- A description of any restrictions on data availability
- For clinical datasets or third party data, please ensure that the statement adheres to our policy

All data are available in the main text or the supplementary materials.

Human research participants

Policy information about studies involving human research participants and Sex and Gender in Research.

Reporting on sex and gender	This study did not involve human research.
Population characteristics	This study did not involve human research.
Recruitment	This study did not involve human research.
Ethics oversight	This study did not involve human research.

Note that full information on the approval of the study protocol must also be provided in the manuscript.

Field-specific reporting

Please select the one below that is the best fit for your research. If you are not sure, read the appropriate sections before making your selection.

[x] Life sciences [ ] Behavioural & social sciences [ ] Ecological, evolutionary & environmental sciences

For a reference copy of the document with all sections, see nature.com/documents/nr-reporting-summary-flat.pdf

Life sciences study design

All studies must disclose on these points even when the disclosure is negative.

Sample size

1. To examine the cleavage pattern of NcVg, protein was extracted in equal amounts from the ovaries of 30 female leafhoppers, as well as the whole bodies or salivary glands from 30 nonviruliferous or viruliferous leafhoppers.
2. To investigate the release of salivary proteins in rice plants, approximately 50 adult leafhoppers were allowed to feed on a single rice seedling.
3. To detect the release of target proteins to rice plants using immunofluorescence microscopy, 15 female adults were allowed to feed on a region of a single rice seedling in a small cage.
4. To trace target proteins of leafhoppers in rice plants, approximately 30 viruliferous or nonviruliferous leafhoppers were allowed to feed on a region of a single rice seedling.
5. To determine the RNAi efficiency, RT-qPCR and western blot assays were performed on salivary glands from 30 dsRNA-treated leafhoppers.
6. To test the presence of target proteins in plant samples using western blot assays, approximately 50 dsRNA-treated leafhoppers were allowed to feed on one rice seedling.
7. For the relative quantification of target gene expression in salivary glands, total RNAs were extracted from the salivary glands of leafhoppers (nonviruliferous, viruliferous, dsGFP-treated, or dsNcVg-treated) obtained from 30 leafhoppers.
8. To test the content of JA, SA and H2O2, as well as related metabolites, approximately 30 female adults were starved for 2 hours and then were allowed to feed on 1 rice seedling.
9. To examine the relative expression of genes related to JA, SA and ethylene, 30 leafhoppers (nonviruliferous or viruliferous) were fed on 1 rice seedling.
10. To visualize of H2O2 location and accumulation, 5 female adults were starved for 2 hours and then fed on a region of 1.5 cm in length by 0.3 cm in width of 1 leaf of rice seedling at the 2-leaf stage in a small cage for 12 hours.
11. To test the effect of NcVg knockdown of viruliferous leafhoppers on insect resistance of rice and viral transmission, salivary glands of 30 dsGFP- or dsNcVg-treated viruliferous leafhoppers were dissected and analyzed in RT-qPCR and western blot assays. Thirty dsGFP- or dsNcVg-treated nonviruliferous or viruliferous leafhoppers were fed on 1 rice seedling, which were then analyzed using western blot assays. Fifty dsGFP- or dsNcVg-treated nonviruliferous or viruliferous leafhoppers were fed on 1 rice seedling, which were then determine the contents of rice H2O2, MDA, GSH, and GSSG, as well as the activities of GST, POD, and CAT.
12. To conduct EPG technique, each leafhopper was continuously recorded for 3 hours. Thirteen a valid biologically independent replicates were recorded.
13. To test the effect of RDV infection on NcVg release into rice plants, salivary glands from 30 viruliferous or nonviruliferous leafhoppers were dissected and analyzed using RT-qPCR and western blot assays.
14. To test the effect of GW4869 treatment on NcVg release from salivary glands, salivary glands from 30 GW4869-treated leafhoppers were analyzed in western blot assays. Approximately 50 GW4869- or DMSO-treated leafhoppers were allowed to feed on 1 rice seedling.
15. To test the activity of POD and CAT, as well as the contents of H2O2 and MDA in NcVg2-OE plants and WT plants at tillering stage, approximately 30 leafhoppers were allowed to feed on 1 leaf. To visualize H2O2 location and accumulation in NcVg2-OE plants, 5 leafhoppers were allowed to feed on a region 1.5 cm in length by 0.5 cm in width of 1 leaf of NcVg2-OE plants and WT plants at booting stage for 12 hours.
16. To test GST activity, GSH and GSSG contents, approximately 30 female adult leafhoppers (nonviruliferous, viruliferous) were starved for 2 hours and then were allowed to feed on a region of 1 leaf of WT or NcVg2-OE plants for 12 hours.
17. To determine the effect of NcVg knockdown on OsGSTF12 suppressing H2O2, approximately 30 dsNcVg- or dsGFP-treated leafhoppers were allowed to feed on 1 leaf of rice plants.
18. To examine the effect of NcVg knockdown on the suppression of H2O2 by OsGSTF12, approximately 30 dsNcVg- or dsGFP-treated leafhoppers at 4 days post-microinjection were allowed to feed on 1 leaf of rice plants.
19. To determine contents of GSH and GSSG in OsGSTF12-KO plants, approximately 30 leafhoppers were allowed to feed on a region of 1 leaf of OsGSTF12-KO and WT plants.

	20. Approximately 50 viruliferous newly emerged female adults were starved for 2 hours, then fed on 1 leaf of NcVg2-OE, OsGSTF12-KO, or WT plants.
Data exclusions	No data were excluded from the analyses.
Replication	All experiments of RT-qPCR, western blot, contents of JA, SA H2O2, MDA, GSH, and GSSG, as well as the activities of GST, POD, and CAT were performed for at least 3 biological replicates. All EPG recorded at least 13 valid biologically independent replicates. Fifteen random 20×20 µm fields of samples from infected or uninfected salivary glands were examined in immunofluorescence microscopy. Fifteen random samples from infected or uninfected salivary glands were examined in immunoelectron microscopy. The number of feeding holes per cm^2 of leaves from 5 biological replicates were examined.
Randomization	Leafhopper or rice samples were randomly collected for experimental groups.
Blinding	We were blinded to group allocation during data collection and analysis.

Reporting for specific materials, systems and methods

We require information from authors about some types of materials, experimental systems and methods used in many studies. Here, indicate whether each material, system or method listed is relevant to your study. If you are not sure if a list item applies to your research, read the appropriate section before selecting a response.

Materials & experimental systems

n/a	Involved in the study	
☐	☒	Antibodies
☒	☐	Eukaryotic cell lines
☒	☐	Palaeontology and archaeology
☐	☒	Animals and other organisms
☒	☐	Clinical data
☒	☐	Dual use research of concern

Methods

n/a	Involved in the study	
☒	☐	ChIP-seq
☒	☐	Flow cytometry
☒	☐	MRI-based neuroimaging

Antibodies

Antibodies used	Rabbit polyclonal antisera against RDV antigens was provided by Dr. Toshihiro Omura of the National Agricultural Research Center, Japan. Polyclonal antibodies against P8 were obtained from ABclonal, China, while polyclonal antibodies against NcRab27a and NcRab5 were sourced from Beyotime, China. Genscript Biotech Corporation, Nanjing, China, prepared polyclonal antibodies against NcVg2 and OsGSTF12, and the process was approved by the Science Technology Department of Jiangsu Province of China.
Validation	Genscript Biotech Corporation, Nanjing, China, prepared polyclonal antibodies against NcVg2 and OsGSTF12, and the process was approved by the Science Technology Department of Jiangsu Province of China.

Animals and other research organisms

Policy information about studies involving animals; ARRIVE guidelines recommended for reporting animal research, and Sex and Gender in Research

Laboratory animals	Rice green leafhopper Nephotettix cincticeps.
Wild animals	Nonviruliferous individuals of N. cincticeps were collected from rice fields in Fujian Province, southeastern China.
Reporting on sex	Not applicable.
Field-collected samples	Nonviruliferous individuals of N. cincticeps were collected from rice fields in Fujian Province, southwestern China, and propagated for several generations in the laboratory. The initial source of RDV-infected rice plants was also collected from rice fields in Fujian Province and propagated via transmission by N. cincticeps under greenhouse conditions.
Ethics oversight	No ethical approval or guidance was required because the materials were insects and plant.

Note that full information on the approval of the study protocol must also be provided in the manuscript.
